# Confidence in Adherence to Antidepressant Prescribing Guidelines Among Liaison Psychiatrists

**DOI:** 10.1192/bjo.2024.93

**Published:** 2024-08-01

**Authors:** Lois Zac-Williams, Gareth Smith

**Affiliations:** 1 Barking, Havering and Redbridge Univeristy Hospitals NHS Trust, London, United Kingdom; 2 North East London NHS Foundation Trust, London, United Kingdom

## Abstract

**Aims:**

This project aims to increase confidence among Liaison Psychiatrists (LPs) in North East London Foundation Trust (NELFT) regarding their adherence to the prescribing guidelines for antidepressants by 25% in accordance with the standard set by Psychiatric Liaison Accreditation Network (PLAN).

Background The prescribing guidelines in this project are based on Standard 21 from 7^th^ Edition Standards as devised by PLAN which states:

“When medication is prescribed, specific treatment goals are set with the patient, the risks (including interactions) and benefits are discussed, a timescale for response is set and patient consent is recorded.”

This project focuses on antidepressants because they are one of the widely used medications in psychiatry that doctors of all grades working in Liaison Psychiatry will be familiar with to some extent. Adhering to this validated guideline would promote gaining informed consent and patients’ involvement in their care, which studies have shown can increase adherence to treatment.

**Methods:**

Circulated an eight-question survey by email based on Standard 21 of 7^th^ Edition Standards document by PLAN to LPs in NELFT. Conducted two Plan-Do-Study-Act (PDSA) cycles. The first PDSA uses a teaching session as the intervention and explained the importance of antidepressant guidelines and what areas LPs need to address with patients. The second intervention uses a poster to reinforce the key points. After each intervention a reissued survey assesses the change in responses.

**Results:**

The baseline survey response rate was 10 out of 15 LPs, made up of seven consultants, two registrars and one foundation year doctor. The lowest levels of confidence were reported around providing patients with printed information on their prescribed antidepressant with the majority of consultants reporting the lowest level of confidence. The highest levels of confidence across all medical grades were reported around discussing a specific treatment goal and explaining the benefits of treatment with antidepressant medication.

**Conclusion:**

From the baseline data, it can be concluded that providing patients with printed information on newly prescribed antidepressants is the area that LPs, particularly consultants, are the least confident about regarding their adherence to prescribing guidelines. Future cycles of this quality improvement project can assess how incorporating teaching on antidepressant prescribing guidelines into trust induction sessions impacts LPs confidence in their adherence.

